# Editorial: Disease-on-a-chip: from point-of-care to personalized medicine

**DOI:** 10.3389/fphar.2023.1344379

**Published:** 2023-12-11

**Authors:** Andrea Cruz, Elisabete Fernandes, Raquel O. Rodrigues, Susana O. Catarino, Diana Pinho

**Affiliations:** ^1^ International Iberian Nanotechnology Laboratory (INL), Braga, Portugal; ^2^ Microelectromechanical Systems Research Unit (CMEMS-UMinho), School of Engineering, University of Minho, Guimarães, Portugal; ^3^ LABBELS—Associate Laboratory, Braga, Portugal

**Keywords:** lab-on-chip, microfluidic devices, miniaturization, organ-on-chip, detection systems, drug-device, digital pills

## Introduction

**FIGURE 1:**
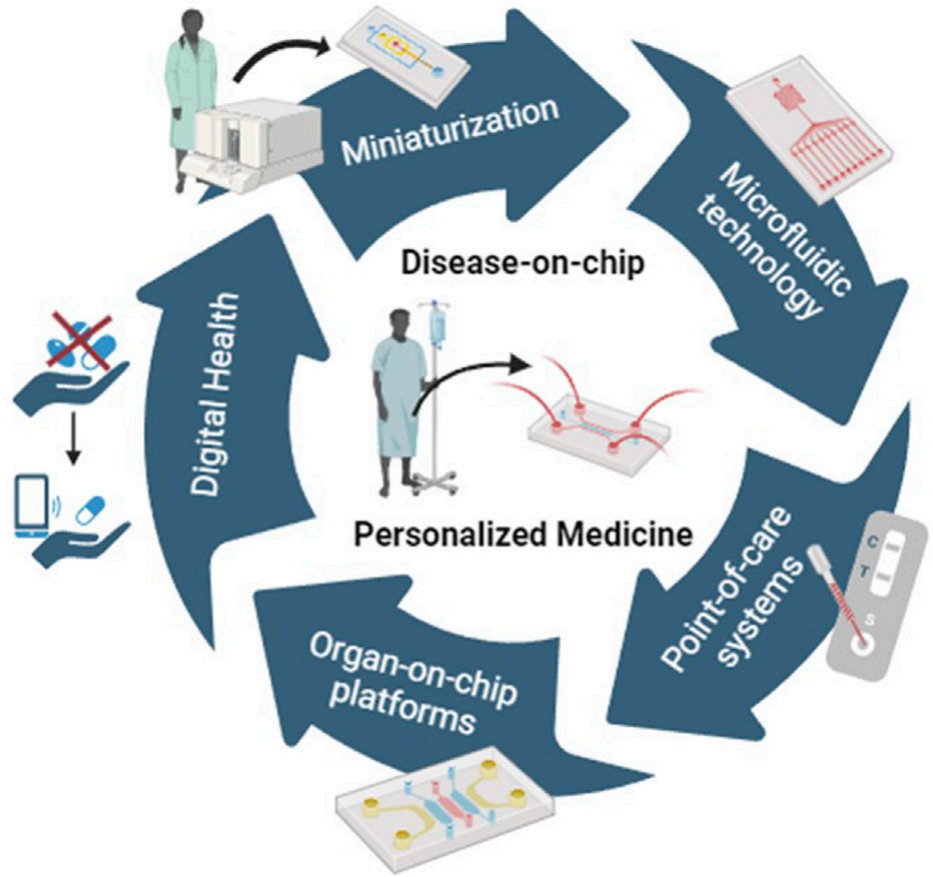
Integration of cutting-edge technologies to innovative developments in the field of personalized medicine. Special issue “Disease-on-chip: From point-of-care to personalized medicine”.

Miniaturization has played a pivotal role in driving innovation in microfluidic devices, particularly in their application across advanced technologies, such as Lab-on-chip platforms, Point-of-care systems, Organ-, Body- and Disease-on-a-chip technology. The personalized nature inherent in these systems, coupled with physiologically relevant read-outs, opens novel avenues for individualized disease diagnostics, Figure 1. Additionally, they offer personalized strategies for assessing treatment efficacy and safety. Furthermore, the advancement in micro- and nano-microfluidic fabrication allows parallel analysis of large samples sets and the evaluation of different parameters such as biological molecules, cells or drugs. This advancement also facilitates real-time monitoring, thereby enhancing the identification of drug responses.

The integration of cutting-edge microfluidic technologies (Shanti et al.; Dai et al.; Sunildutt et al.), compact detection systems (Chen et al.), and advanced digital medicine platforms (Litvinova et al.) has given rise to innovative tools capable of detecting disease biomarkers through the analysis of a single drop of a body fluid. This breakthrough enables rapid assessment within the timeframe of a doctor’s visit, presenting a valuable feature that supports earlier identification of conditions and facilitates the implementation of personalized and more effective therapeutic options.

For point-of-care diagnostic testing systems, microfluidic devices prove to be a promising technology for sample preparation, offering self- and hand-operated capabilities due to several advantageous properties such as low-sample volume, biocompatibility, low-cost materials and fabrication, easy disposal, and straightforward operation. Chen et al. demonstrated the potential of a paper-based microfluidic device to rapidly and sequentially measure IL-6 concentrations, allowing for early clinical risk assessment and severity stratification of elderly patients with pneumonia (Chen et al.). This represents a potential point-of-care diagnostic device applicable to healthcare settings. Microfluidic platforms designed for fluid or particle separation in downstream analysis have played a significant role in discovering new drug carriers for cancer studies (Dai et al.) and exploring novel approaches for cancer diagnostics and prognostics analysis.

The limitations of animal models in predicting therapeutic responses in humans is a significant challenge, questioning their utility for research. Beyond being costly, time-consuming and ethically questionable, these limitations highlight the need for alternatives approaches. Such alternatives encompass sophisticated *in vitro* assays utilizing human cells and tissues, advanced computer modelling techniques (*in silico* models), and clinical studies. A wide range of new devices are now available, capable to recreate organ-level structures, including tissue–tissue interfaces. These devices also provide relevant biophysical, biochemical, mechanobiological, and mechanical cues, such as simulating breathing and peristalsis-like motions, essential for faithfully modelling organ physiology and disease states (Sunildutt et al.).

By fluidically coupling two or more organ chips, human ‘body-on-chips’ multi-organ systems can be created mimicking whole-body physiology, as well as, drug distribution and disposition. Sunildutt et al., provide a comprehensive review of essential parameters for developing an organ-on-chip platform to mimic diseases, genetic disorders, access drug toxicity effects in different organs, identify biomarkers, and aid in drug discovery. Parameters as sheer stress, flow rate, cell type, platform material and design are crucial aspects for accurate *in vitro* disease modelling. Despite advancements, several challenges persist in the acceptance of organ-on-chip platforms as standard pre-clinical model tools by drug regulatory agencies and pharmaceutical industries (Sunildutt et al.).

An example of a disease-on-chip is the lymph node on-chip, which incorporates multiple design elements, microfluidic pathways, 3D cellular matrix, co-culture of multiple cell types, chemotaxis and cellular communication. All these elements supported within a biologically sustainable microfluidic microenvironment that make the lymph node device a biologically relevant tool for pharmacological and toxicological applications (Shanti et al.).

Digital pills represent a novel technological advancement that aim to improve healthcare by optimizing medication administration. These innovative drug-device technologies integrate traditional medications with a monitoring system, allowing the recording of data on medication adherence, as well as patients’ physiological data without the need for direct human intervention, such as through ingestible sensors. Litvinova et al. demonstrate the evidence that this new area of digital medicine can access high effectiveness, safety, and prospects for the future medicine in mental health, tuberculosis, cardiovascular disease, uncontrolled hypertension, and type 2 diabetes; tuberculosis, hepatitis C, and AIDS, among others (Litvinova et al.). This digital pills are increasingly viewed as an acceptable, practical, and beneficial technology within the medical system.

The incorporation of these technologies into medicine practice is undeniably crucial for overcoming challenges such as missed diagnoses, delayed treatment, and poor adherence to prescribed treatments, preventing disease progression, disability, and, in some cases, death. Thus, improvements in healthcare systems can be obtained, towards a medicine that is increasingly predictive, preventive, personalized and participatory.

## Future opportunities and challenges

Lab-on-a-chip technologies involve the use of microengineered devices, and a critical aspect of their development lies not only in adhering to engineering specifications and principles but also in ensuring scalability, manufacturability, reproducibility, and accuracy. The evolution of manufacturing technologies, optimizing conventional micro- and nano-lithographic methods, and integrating additive manufacturing methods like 3D-printing, is expanding the fabrication and applicability of microfluidic devices.

Beyond lab-on-a-chip applications, the field of intelligent microfluidics is emerging, incorporating machine learning for monitoring and controlling microfluidic systems. This presents new challenges and opportunities to develop novel molecular signatures, the synthesis of new therapeutic drugs and targets, and advancements in materials’ discovery.

Along with the integration of machine learning in the medicine, innovative drug-device technologies, such as digital pills combining traditional medication with intelligent monitoring systems, lay the foundation for the future medicine. This concept defines a new generation of the Digital Medicine System that holds the potential to transform unsustainable healthcare systems.

However, and as abovementioned, the implementation of personalized disease-on-chip systems faces a significant challenge from medical and pharmaceutical communities, regulatory bodies, and government agencies, primarily due to ethical concerns surrounding health data. Therefore involving all biomedical stakeholders from clinicians and patients to pharmaceutical companies, becomes crucial for the successful transition of personalized disease-on-chips to precision medicine.

Collaborative efforts and ethical frameworks will be instrumental in addressing these challenges and unlocking the full potential of microfluidic technologies in advancing healthcare.

